# Assessing the Clinical Feasibility of the DiaFocus System for Integrated Personalized Management of Type 2 Diabetes: 6-Month Pilot Cohort Study

**DOI:** 10.2196/63894

**Published:** 2025-08-25

**Authors:** Nanna Lind, Per Bækgaard, Jakob E Bardram, Claus Cramer-Petersen, Kirsten Nørgaard, Merete B Christensen

**Affiliations:** 1 Copenhagen University Hospital – Steno Diabetes Center Copenhagen, Borgmester Ib Juuls vej 83, Herlev, 2730, Denmark, 45 28711435; 2 Department of Clinical Medicine, Faculty of Health and Medical Sciences, University of Copenhagen, Copenhagen, Denmark; 3 Department of Applied Mathematics and Computer Science, Technical University of Denmark, Lyngby, Denmark; 4 Department of Health Technology, Technical University of Denmark, Lyngby, Denmark; 5 Coloplast A/S, Humlebaek, Denmark

**Keywords:** mHealth, mobile health, digital health system, digital platform, type 2 diabetes, diabetes mellitus, diabetes management, feasibility, pilot study, shared decision-making, chronic condition, monitoring, communication, adult, Denmark, diabetes treatment, diet, exercise, medication, smoking, stability

## Abstract

**Background:**

Type 2 diabetes (T2D) is a complex, chronic condition that requires ongoing management. An important aspect of effective diabetes management is shared decision-making between the person with diabetes and the health care professionals (HCPs) to tailor individual treatment plans. Personal health technologies can play a crucial role in this collaborative effort by providing tools for monitoring, communication, and education.

**Objective:**

This study aims to test the clinical feasibility of DiaFocus, a mobile health system developed for adults with T2D.

**Methods:**

This was a single-arm, prospective, 6-month pilot study in a clinical outpatient setting at Steno Diabetes Center Copenhagen, Denmark. The DiaFocus system includes an app for the participants and a web portal for the HCPs. The system collects diabetes-related data, including participant-reported lifestyle surveys, sensor-based measures on physical activity, and participant-selected focus areas, aiming to support communication and shared decision-making at clinical visits. Participants were eligible if they were ≥18 years old, diagnosed with T2D≥12 months, spoke Danish, and had a smartphone (iOS 13+ or Android 8.0+). For each participant, 3 visits and 1 telephone call were scheduled during the 6-month study period. The DiaFocus system’s acceptability and feasibility were assessed through retention rates, app usage, participant feedback, and by the CACHET Unified Method for Assessment of Clinical Feasibility (CUMACF) questionnaire. The clinical outcomes were assessed by the following questionnaires: Diabetes Distress Scale (DDS), Perceived Competence for Diabetes (PCDS), Diabetes Treatment Satisfaction Questionnaire (DTSQs+c), hemoglobin A_1c_ levels, and body weight.

**Results:**

A total of 17 participants with T2D were included in the study, 15 completed the study, and data were analyzed on an intention-to-treat basis. The median age was 68 (IQR 56-72) years, 12 (71%) were males, the median diabetes duration was 18 (IQR 11-21) years, and the median hemoglobin A_1c_ was 59 (IQR 49-68) mmol/mol. Participants found the DiaFocus system feasible to support diabetes management despite technical problems, and they valued the ability to set focus areas. The most common focus areas were “blood glucose” (n=10, 59%) and “exercise” (n=9, 53%), but areas such as “sleep” and “mood” were also used. The CUMACF questionnaire showed that 90% (9/10) of the participants had very favorable views of how easy the system is to understand, learn, and use, and 80% (8/10) of the participants agreed or strongly agreed that the system was useful. Feedback was generally positive, indicating participants would use a refined version. Despite these findings, no statistically significant changes in clinical outcomes were observed throughout the study period using the DiaFocus system.

**Conclusions:**

This pilot study demonstrated that the DiaFocus system is clinically feasible and acceptable for users with T2D, although there is a need for optimization of app functionality and stability.

## Introduction

### Background

Type 2 diabetes (T2D) is a chronic disease that affects many aspects of daily life, and managing diabetes can be challenging for individuals with T2D. It is well established that optimal disease management can lead to fewer diabetes-related complications and decreased mortality [1,2]; yet, many struggle to reach diabetes treatment goals [3]. Medical treatment of T2D includes a wide range of oral and injectable therapeutics. However, factors beyond medication can impact blood glucose levels, such as stress, physical activity, concomitant diseases, and sleep disturbances [4-6]. Accordingly, diabetes self-management education and support are crucial parts of optimal diabetes management, involving a combination of lifestyle modifications, medication management, and self-care strategies [7]. Personal health technology has been highlighted as a possible way to improve T2D self-management by providing more personalized, data-driven care. In addition, it has the potential to empower individuals with T2D to gain deeper insight into and manage their condition more efficiently, while also supporting collaboration between the health care professional (HCP) and the individual with diabetes [8,9].

### Prior Work

An integrated personalized diabetes management (iPDM) approach has previously been described [10,11]. In short, the iPDM approach consists of a structured, 6-step process that uses digital tools and aims to support collaborative diabetes care and shared therapeutic decision-making. Using this approach, the iPDM-ProValue study program, which included 907 persons with insulin-treated T2D in Germany, demonstrated improved glycemic control and treatment satisfaction [11]. A secondary analysis from the iPDM-ProValue study program further demonstrated that both patients and HCPs perceived a benefit in using digital tools in a structured manner [12].

DiaFocus is a novel smartphone-based system based on the principles of the iPDM approach [13]. The DiaFocus system includes an app for the participants and a web portal for HCPs. The system is designed to collect diabetes-related data, including participant-reported outcomes, sensor-based measures on physical activity, and participant-selected focus areas, aiming to support communication and shared decision-making between the HCP and the person with diabetes. The technical feasibility of using the DiaFocus app for T2D management was assessed in a 6-week pilot study with 12 participants [13]. This study showed that participants found the DiaFocus approach and system useful and usable for diabetes management and that most patients would use such a system, if available, as part of their treatment. Feedback from the participants in this study was used to adjust the system.

### Goal of This Study

In this clinical pilot study, we aimed to test the clinical feasibility of the DiaFocus system and to provide preliminary evidence of effectiveness. The study was conducted in a clinical outpatient setting for 6 months.

## Methods

### Study Design

This clinical pilot study was a single-arm, prospective, 6-month cohort study. We used a convergent study design (mixed methods approach) where quantitative and qualitative data were collected to explore the participants’ experience using a shared decision solution containing an app for the participants and a web portal for HCPs.

### Study Participants and Recruitment

Participants were recruited from the outpatient clinic at Steno Diabetes Center Copenhagen (SDCC) in Denmark by their HCPs. Participants were preselected based on inclusion criteria. Participants were eligible if they were ≥18 years, diagnosed with T2D more than 12 months ago, treated at the outpatient clinic at SDCC for at least 12 months, understood and spoke Danish, and had a smartphone (with an operating system of at least iOS version 13 for Apple devices and version 8.0 Oreo for Android devices). Exclusion criteria were severe visual impairment or other conditions not compatible with participation (judged by the investigators). For persons interested in study participation, a subsequent consultation was scheduled and spoken and written information was given by the investigators.

### System Description

**Figure 1.**
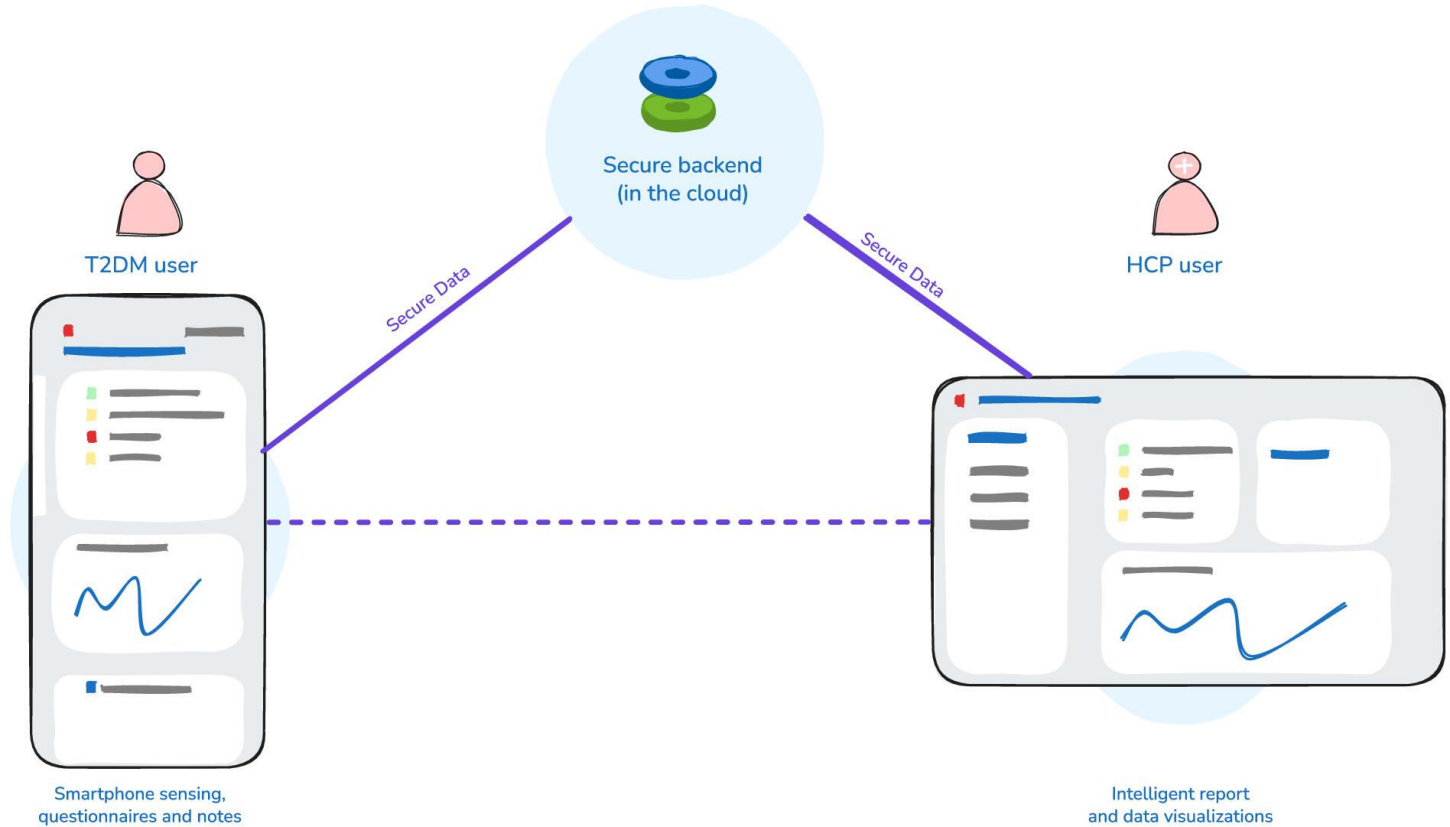
The DiaFocus system. HCP: health care professional; T2DM: type 2 diabetes mellitus.

The DiaFocus system used to support the iPDM cycle [10,11] consists of a mobile app used by the participant and a web portal used by the HCPs (Figure 1), sharing data via a secure backend server [13].

**Figure 2.**
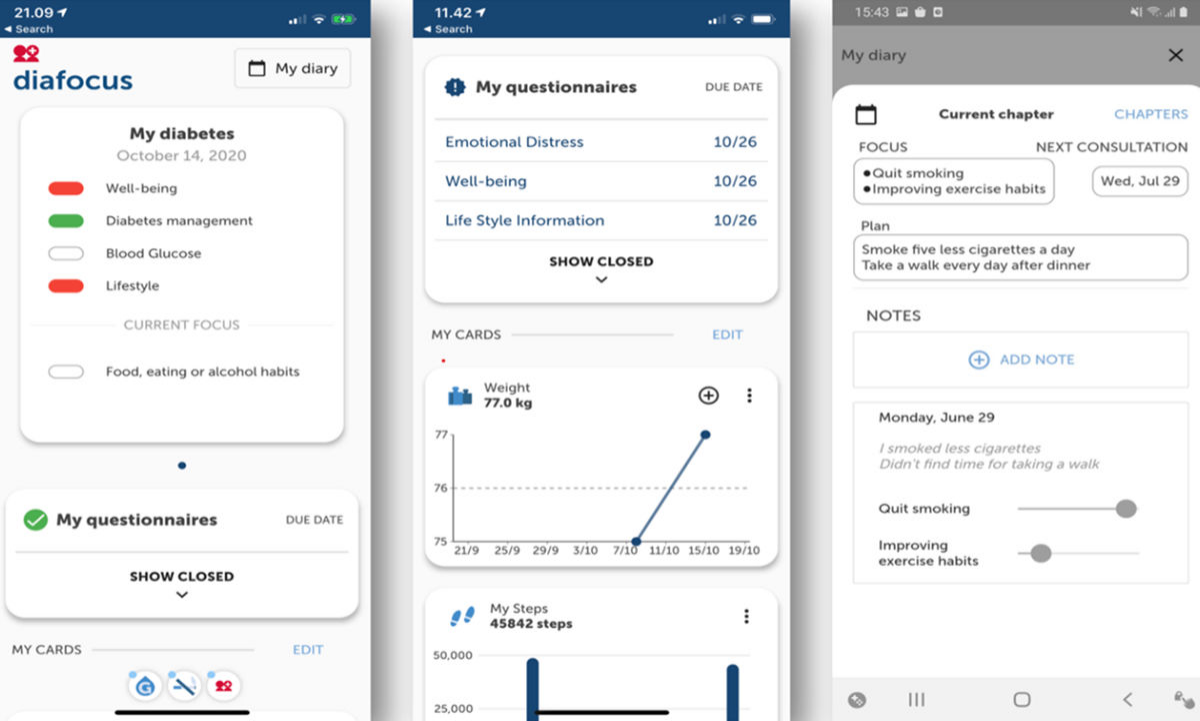
The user interface of the DiaFocus app.

The DiaFocus app was installed on the participant’s own smartphone and used for collecting three types of data: (1) patient-reported health and lifestyle measures such as blood glucose level, smoking, and alcohol consumption; (2) standardized questionnaires such as the World Health Organization Well-being Index (WHO-5) [14]; and (3) automatic collection of sensor data from the phones onboard sensors, such as the step counter. The type of lifestyle measures and questionnaires was adapted to the focus areas of the patient, an approach called “adaptive assessment,” which worked in the following manner. All participants were prompted to answer the WHO-5 questionnaire and 2 questions on problems with diabetes. Depending on the answers hereof, additional questionnaires on sleep, anxiety, and depression were triggered. At each visit to the diabetes clinic, the participants created a new chapter and registered one or more focus areas. The participants could choose as many focus areas as they wanted and had the opportunity to continue or change focus areas at each visit. Some focus areas were prespecified (blood glucose measurements, diet, activity, and smoking); however, participants could also add a focus area of their own. The participants were subsequently able to track progress related to this area; if the participant and HCP, for example, decided to focus on reducing smoking, the participant could subsequently track and monitor daily smoking habits. At the next clinical visit, data collected through the phone, including responses to agreed questionnaires, were then aggregated and shown as a report on the web portal, which formed the basis for the following discussion and renewed focus area and target setting. The design of the DiaFocus system and the “adaptive assessment” approach are described in more detail elsewhere [13]. An example of a smartphone screen and part of a web portal report is shown in Figures2  3. Figure 2 shows the health status (left), the list of pending questionnaires and data visualization (middle), and the editing of a chapter (right).

**Figure 3.**
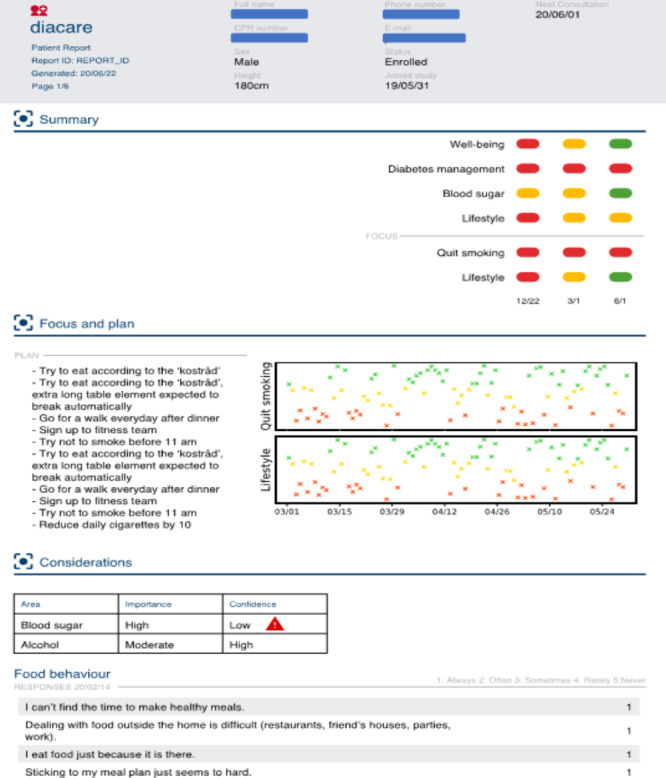
Example of a patient report from the web portal.

### Study Procedures

Three visits and one telephone call were scheduled for each participant during the study period. The 3 visits were carried out in extension of the participants’ regular clinical visits at baseline (visit 1), after 3 months (visit 2), and after 6 months (visit 3) according to the iPDM process. The 6-month study period allowed for the evaluation of 2 iPDM cycles, enabling adjustment of the process individually if needed.

At the baseline visit, participants were screened for eligibility, and after giving oral and written informed consent, initial baseline data were collected. The DiaFocus app was installed on the participant’s own smartphone, the app was introduced to the participant, initial focus areas were set up for the participant, and the baseline questionnaires were completed. The participants were encouraged to use the app regularly at home. A telephone call was scheduled 2 weeks after the participants’ first visit. The purpose of the call was to ensure that the app was operational and that the participants understood how to use the app. At visits 2 and 3, the HCP reviewed and assessed the data uploaded from the app together with the participant. Accordingly, the individual diabetes management plan was adapted, and future focus areas were planned through shared decision-making.

Blood samples were collected to analyze hemoglobin A_1c_ (HbA_1c_), blood glucose, creatinine, estimated glomerular filtration rate, and lipid profiles at baseline, and a urine sample was analyzed for albumin/creatinine ratio. HbA_1c_ level was measured at visits 2 and 3. Participant-reported outcomes were measured at baseline and visit 3 using the following questionnaires: Diabetes Distress Scale (DDS) [15-17], Perceived Competence for Diabetes (PCDS) [18,19], and Diabetes Treatment Satisfaction Questionnaire (DTSQs+c) [20].

### Outcome Measures

#### Clinical Feasibility

Assessment of the acceptability and feasibility of the DiaFocus system was measured by retention of participants in the study, the use of the DiaFocus app, willingness to share selected focus areas, and feedback from the participants during and after the study. While behavioral changes were primarily captured through self-report and usage data, these were not assessed using blinded evaluators, given the nature of the study and its feasibility design. To assess the perceived clinical feasibility of the DiaFocus system, participants who had indicated they would accept being contacted after the last visit were asked to respond to the CACHET Unified Method for Assessment of Clinical Feasibility (CUMACF) questionnaire [21]. The CUMACF questionnaire measures a system’s perceived usefulness and usability if the tested system were to become available to the participants. As a result, any technical issues or glitches particular to the present implementation therefore receive less emphasis. It is overall divided into five sections, measuring (1) Health Expectancy (do the participants perceive using the system could help attain health benefits), (2) Effort Expectancy (would the system be perceived to be easy to use), (3) Social Influence (how important would others be expected to find it to be using the system), (4) Facilitating Conditions (would the needed organizational and technical infrastructure be in place for using the system), and (5) Behavioral Intention (would the participants intend to use the system). The degree to which the participants agree or disagree with each statement is a measure of their expectations toward the system. A Danish version of the questionnaire was used with the study participants; the equivalent English version (Table S2) can be found in the Multimedia Appendix 1. In addition to the CUMACF questionnaire, the participants were also invited to give feedback and comments, including suggestions for future versions of the app. This took place as part of a follow-up phone call after all participants had completed the study. One of the researchers (PB) in random order contacted the 12 participants who had expressed interest in being contacted subsequently. One did not respond to multiple calls, and another excused themselves. The remaining 10 participants replied to the CUMACF questionnaire and additionally provided comments and feedback. No call lasted more than 1 hour. Participants’ feedback and comments were reviewed and grouped into broader categories to summarize key areas according to the overall themes addressed in the CUMACF questionnaire.

#### Clinical Efficacy

The efficacy of the DiaFocus system was assessed by participant-reported outcomes. The primary clinical outcome was a change in diabetes-related distress from baseline to visit 3 (measured by the DDS). DDS includes a focus on self-management and physician-related distress. DDS consists of 17 items on a 6-point scale [15-17]. Secondary outcomes were change in PCDS, change in DTSQs+c (Table S1 in Multimedia Appendix 1), and change in HbA_1c_ levels and body weight.

### Statistical Analyses

Due to the nature of the study being a feasibility design, no sample size was calculated. We aimed to assess feasibility and gather preliminary data to inform the design of a future, larger-scale trial. We chose a recruitment window of 12 months and were able to include 17 participants. This number was considered adequate to assess our feasibility objectives. Analyses comparing changes from baseline to end of study were performed using the *t* test for parametric data and Wilcoxon rank-sum test for nonparametric data. The chi-square or Fisher exact test was used for categorical data. The analyses followed the intention-to-treat principle, and no adjustments were made for multiple comparisons.

All statistical analyses were done using SAS Enterprise Guide version 8.3 (SAS Institute Inc). Data are presented as median (IQR) or mean (SD) unless otherwise stated. A 2-sided *P* value of ≤.05 was considered statistically significant.

### Ethical Considerations

The study was carried out in accordance with the Declaration of Helsinki after approval by the Regional Scientific Ethics Committee (H-20040944) and the data protection agency (P-2020‐1055). Informed consent was obtained from all participants before participation. Written patient information was given along with the brochure: “Forsøgspersoners rettigheder i et sundhedsvidenskabeligt forskningsprojekt” (Rights of study subjects in a health science research project). The investigator ensured that the potentially eligible participants were adequately informed about the study rationale and design, in written and spoken words. Before signing the consent form, the person was given a minimum of 24 hours to reconsider. Potentially eligible participants were informed that they, at any time, could withdraw their informed consent without having consequences for their future treatment. All information on study participants was protected according to the law on the processing of personal data and the law of health. Data were kept using REDCap (Research Electronic Data Capture; Vanderbilt University), a secure web-based app designed to support research data capture. All personally identifiable information on paper was kept in a locked filing cabinet in a double-locked office. All data were deidentified before any data analysis. The study participants did not receive any compensation for being in the study.

## Results

From February 2021 to April 2022, a total of 18 potential eligible participants with T2D were screened. There was one screen failure due to severe nephropathy not compatible with study participation. Two participants dropped out during the study due to technical problems with the app; accordingly, 15 completed the study, and data from all 17 participants were analyzed. The median age was 68 (IQR 56-72) years, 12 (71%) were males, the median diabetes duration was 18 (IQR 11-21) years, and 10 (59%) were treated with insulin. The HbA_1c_ level was 59 (IQR 49-68) mmol/mol. Additional baseline characteristics are described in Table 1.

**Table 1.** Baseline characteristics of the included participants.

Baseline characteristics	All participants (N=17)
Age (years), median (IQR)	68 (56-72)
Gender (male), n (%)	12 (71)
Race (White), n (%)	17 (100)
BMI (kg/m^2^), median (IQR)	29 (26-32)
Diabetes duration (years), median (IQR)	18 (11-21)
HbA_1c_^a^ baseline (mmol/mol), median (IQR)	59 (49-68)
Diabetes treatment, n (%)	
Metformin	11 (64)
GLP-1^b^ receptor agonist	12 (71)
SGLT2^c^ inhibitor	12 (71)
Basal insulin	6 (35)
Basal-bolus insulin	4 (24)
How often do you measure BG^d^?, n (%)	
Never	2 (12)
1‐4/month	5 (29)
2‐3/week	2 (12)
4‐6/week	1 (6)
1‐2/day	4 (24)
3 or more/day	3 (18)
How often do you send text messages?, n (%)	
1‐3 d/mo	1 (6)
1‐2 d/wk	1 (6)
3‐5 d/wk	1 (6)
6‐7 d/wk	14 (82)
How often do you use other apps on your phone?, n (%)	
1‐3 d/mo	0 (0)
1‐2 d/wk	1 (6)
3‐5 d/wk	0 (0)
6‐7 d/wk	16 (94)

a HbA_1c_: hemoglobin A_1c_.

b GLP-1 receptor agonist: glucagon-like peptide-1 receptor agonist.

c SGLT2 inhibitor: sodium-glucose transport protein-2 inhibitors.

d BG: blood glucose.

### Clinical Feasibility

#### Focus Areas

Participants reported that the feature of setting focus areas was of great value to them, and all wanted to share their choice of focus areas.

They were most likely to choose blood glucose measurement (17 times, for 10 participants), exercise (14 times, for 9 participants), food (8 times, for 5 participants), medication-taking (7 times, 4 participants), or sleep (4 times, for 3 participants) as a focus area during the study. Weight management (1), mood (1), and heart rate (2 times for 1 participant) were also chosen as focus areas.

#### Questionnaires in the App

The questionnaires were planned to be triggered 14 days before the clinical visits. Due to initial technical issues, not all participants received the questionnaires before visits 2 and 3. At baseline, all participants’ median WHO-5 well-being score was 68 (out of 100). Two participants had WHO-5 score ≤50, indicating low emotional well-being. A sleep questionnaire was triggered for 10 out of the 17 participants (59%) during the study. A depression and anxiety questionnaire was triggered for 6 of the participants during the study.

#### CUMACF Questionnaire

**Figure 4.**
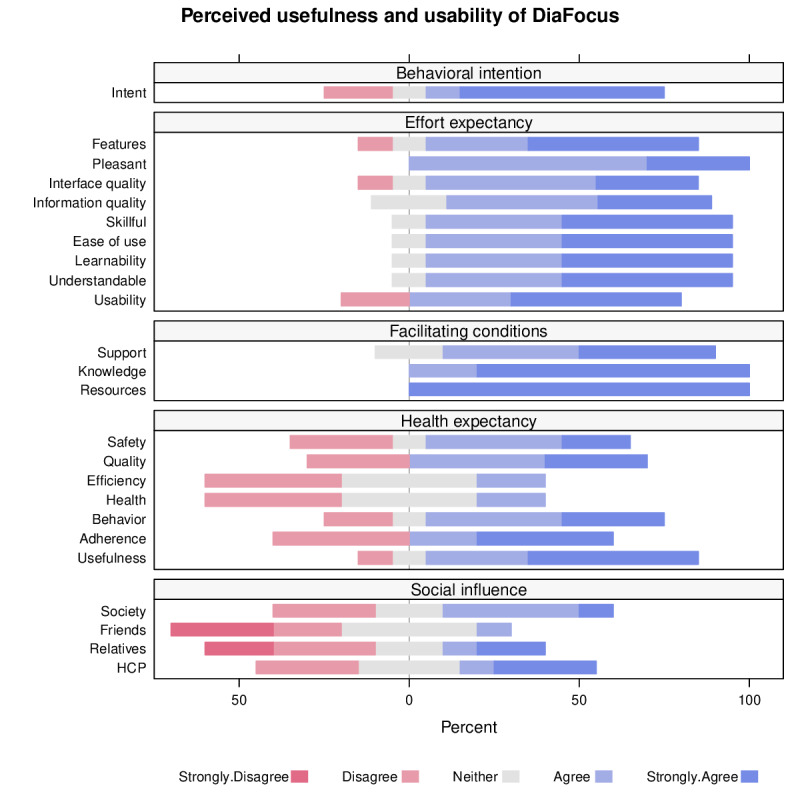
The results of the CUMACF (CACHET Unified Method for Assessment of Clinical Feasibility) questionnaire.

Of the 15 participants who completed the study, 3 had indicated no interest in being contacted later. A total of 10 of the remaining 12 participants were reachable and willing to reply to the CUMACF questionnaire. A summary of their responses is shown in Figure 4.

For each statement, the proportion of respondents strongly disagreeing or disagreeing is shown in red, neither disagreeing nor agreeing in gray, and agreeing or strongly agreeing in blue. Agreeing or Strongly Agreeing to a statement generally indicates a positive response or perception.

Regarding Behavioral Intention, 70% (7/10) of the participants “agree” or “strongly agree” that they would use the system for 3 months if it were available as a service.

Regarding Effort Expectancy, 90% (9 out of 10) participants, for example, have very favorable views of how easy the system is to understand, learn, and use. Furthermore, 80% (8/10) also expect the usability to be high.

Regarding the Facilitating Conditions, participants almost uniformly agree that the necessary conditions would be available for them to use the system; all (10/10) agree they have the technical equipment and knowledge to use the system but 80% (8/10) indicate that they nevertheless expect a technical hotline would be available.

Regarding Health Expectancy, participants are generally positive regarding the overall usefulness, their expected adherence, and behavior; 80% (8/10) agree or strongly agree that the system is useful. Regarding the expected health outcome and efficiency, they are less positive. However, 70% (7/10) agree or strongly agree that the system would improve the quality of the treatment and 60% (6/10) agree or strongly agree that it would reduce the risk of complications.

Participants’ views on Social Influence are mixed.

#### Participant Feedback and Comments

Comments from participants were subsequently reviewed and grouped according to the overall themes addressed using the CUMACF questionnaire. These are summarized below according to (1) perceived usefulness and structure, (2) expectations toward technical integration, (3) overall impact on health outcomes, and (4) finally Social Influence and privacy.

One participant commented that using focus areas and structured follow-up was “helpful to reduce and address concerns” and “help to emphasize the most important elements to handle.” Seven out of the 10 participants (70%) indicated they were willing to use such a system in the future. Others, however, commented that they expected such a system to be “more polished” and “well-integrated with, for example, automatic transfer of blood glucose measurements” to the system.

When asked if the system could potentially reduce complications of their diabetes, 1 participant commented, “…but I already have complications.” Thus, even if the participants were generally positive about the usefulness of the approach, they did not expect that using the system would improve their overall health, as also seen in the lower rating of the efficiency and health scores of the Health Expectancy.

Some participants, when asked about Social Influence from friends and colleagues, commented that they “certainly didn’t involve others” in their diabetes. Another, however, explained that “once his family understood what DiaFocus was about, they strongly encouraged him to use the system.” Hence, diabetes seemed, for many, to be perceived as a private matter, and thus, the Social Influence to use a system like DiaFocus is low.

#### Technical Issues

Ten of the 17 (59%) participants reported technical issues with the app, requiring technical support. During the study, technical problems were identified and fixed on an ongoing basis, and new versions of the software were deployed continuously. Major issues uncovered and reported by participants were impaired step count function, drained phone battery, and unavailable functions after app updates (eg, triggering of questionnaires). In addition, the HCPs reported missing questionnaire data, which complicated the assessment of psychosocial issues. Hence, the participant’s active app use is difficult to report accurately.

### Clinical Efficacy

#### Participant-Reported Outcomes

At baseline, the median DDS score was 1.8 (IQR 1.2-2.2), indicating a low level of diabetes distress, and 1.7 (IQR 1.1-2.4) after 6 months, yielding a median change of 0.18 (IQR 0-0.52; *P*=.18). The total PCDS score was 32 (range 26-32) at baseline and the median change at study end was 1.0 (IQR −3 to 3; *P*=.85). The total DTSQs score was 32 (range 27.5-34) at baseline and remained unchanged after 6 months with a median difference of 0 (IQR 0-1; *P*=.70).

#### HbA_1c_ and Body Weight

The median HbA_1c_ level was 59 (IQR 49-68) mmol/mol at baseline with a median change of 0 (IQR −10 to 3) mmol/mol (*P*=.1) at study end. The median body weight remained unchanged during the study (*P*=.33).

## Discussion

### Principal Findings

This clinical pilot study assessed the use of the DiaFocus system in an outpatient setting for 6 months for adults with T2D. The participants were generally positive about the iPDM treatment approach and found the DiaFocus system feasible to incorporate into their diabetes management. However, no significant clinical changes were seen in participant-reported outcomes, HbA_1c_ level, or body weight. The findings of this study expand upon our previous technical feasibility study [13] by demonstrating how the app can be used in a diabetes clinic to support communication between users and HCPs. Accordingly, participants reported that the feature of setting focus areas and discussing these in the clinic was of great value to them. Sleep was often chosen as a focus area, and a sleep questionnaire was triggered for more than half of the participants. Moreover, a depression and anxiety questionnaire was triggered for one-third of them, indicating a need to address additional health-related areas in diabetes management, including psychosocial issues, during outpatient clinic visits. This need was also emphasized by participants during the interviews, who expressed a desire for a broader focus on diabetes-related health issues beyond just diet, exercise, medication, and smoking.

During the study period, there was a small but insignificant decrease in HbA_1c_ levels. Several previous mHealth intervention studies have demonstrated improved glycemic control; however, most of these interventions also included coaching features, that is, automated messages providing feedback and motivation [9,21], which were not included in the DiaFocus app.

In addition, we did not demonstrate significant changes in participant-reported outcomes (questionnaires DDS, PCDS, and DTSQs). These findings could simply be due to the low number of participants in this pilot study, but the outcomes might also have been influenced by the technical issues with the app. However, despite technical issues with the app, the results from the CUMACF questionnaire indicated that the participants who responded were positive toward the perceived usefulness and usability of a system such as DiaFocus. The concepts were easy to understand and use and were seen as providing value, and many participants would use such a system if it were available to them. These findings are also reflected in the qualitative comments by the participants.

Overall, even if the present implementation experienced technical issues that were only partially resolved during the study, the usability and usefulness of the overall concepts were perceived positively by the participants.

### Limitations

First, a limitation of this study is the feasibility outline, meaning that the study was conducted as a single-arm study with a small sample size without a power calculation. The findings should therefore be interpreted with caution. Second, we included a well-controlled and highly motivated population of individuals with T2D who already had a smartphone. In addition, we did not collect data on socioeconomic status or comorbidities, which may have influenced the outcome of the study. Accordingly, the findings may not be generalizable to other populations. Third, although the CUMACF questionnaire overall shows positive expectations for a system like the one implemented, it could partly result from a sampling bias, as one-third of the participants who were not reachable or willing to reply to a follow-up interview and the CUMACF questionnaire could have been more negative toward the system. Fourth, the absence of a control group limits our ability to definitively attribute any observed improvements to the intervention. Finally, technical difficulties that may have caused some active app usage data to be missing mean that this perspective cannot be properly addressed in the analysis.

### Future Directions

To ensure more accurate and comprehensive results, this feasibility study suggests that future studies should be powered to detect possible significant differences in clinical outcomes. Such studies should also involve larger and more diverse populations to enhance generalizability. This could include individuals with newly diagnosed diabetes, individuals managed in primary care settings, or with higher HbA_1c_ levels. In addition, investigating experiences with the DiaFocus system from a health care provider’s perspective would provide valuable insights and complement the present findings, helping to understand its impact on clinical workflows, communication, and diabetes management. Finally, ensuring a robust technical platform already at the beginning of the study is important to be able to better report and analyze app usage and patients’ engagement with the system throughout the study.

### Conclusions

This pilot study demonstrated that the DiaFocus system was clinically feasible and well accepted among users with T2D, even though no changes were observed in participant-reported outcomes. However, some aspects of the system, including app functionality and technical stability, need optimization before larger and long-term studies are conducted.

## Supplementary material

10.2196/63894 Multimedia Appendix 1 Additional material.

## Abbreviations

CUMACF: CACHET Unified Method for Assessment of Clinical Feasibility
DDS: Diabetes Distress Scale
DTSQ: Diabetes Treatment Satisfaction Questionnaire
HbA_1c_: hemoglobin A_1c_
HCP: health care professional
iPDM: integrated personalized diabetes management
PCDS: Perceived Competence for Diabetes Scale
REDCap: Research Electronic Data Capture
SDCC: Steno Diabetes Center Copenhagen
T2D: type 2 diabetes
WHO-5: World Health Organization Well-being Index
