# Supplementary material for: Assessing the Clinical Feasibility of the DiaFocus System for Integrated Personalized Management of Type 2 Diabetes: 6-Month Pilot Cohort Study
Source: JMIR Diabetes. 2025 Aug 25;10:e63894. doi: 10.2196/63894 (PMC12377514; doi:10.2196/63894)
Supplement: Multimedia Appendix 1 [file diabetes-v10-e63894-s001.docx]

## Supplementary material

**Table S1.** Description of PRO questionnaires

| **Perceived Competence for Diabetes (PCDS)** | The PCDS assesses the degree to which people with diabetes feel they can manage the everyday aspects of diabetes care [18, 19]. The PCDS contains five statements (e.g., ‘I am able to manage my diabetes’), rated on a seven‐point Likert scale indicating a level of agreement (1–7, ranging from ‘not at all true’ to ‘very true’); higher scores represent better respondent's performance. The mean of a person's responses is used as a summary score. |
| --- | --- |
| **Diabetes Treatment Satisfaction Questionnaire (DTSQs+c)** | **DTSQ** is a questionnaire used to assess participants’ satisfaction with their diabetes treatment [20]. The questionnaire consists of a six-item scale assessing treatment satisfaction and two items assessing the perceived frequency of hyper- and hypoglycemia. Each item is scored by participants on a scale ranging from zero (e.g., “very dissatisfied”, “very inconvenient”) to six (e.g., “very satisfied”, “very convenient”). Treatment satisfaction is assessed as the sum of the scores of the six questions on the first factor (total score 36), with a higher score indicating higher treatment satisfaction. The questionnaire is divided into a status questionnaire (DTSQs) for baseline characteristics and a change questionnaire (DTSQc) for intervention end-point measurements. |

**Table S2.** The CUMACF Questionnaire (English version)

| **Category** | **Short name** | **Statement** |
| --- | --- | --- |
| **Health Expectancy** | Usefulness | In general, I would think that DiaFocus is useful for managing my diabetes. |
|  | Adherence | I would use DiaFocus as often as recommended, i.e., daily. |
|  | Behavior | Using DiaFocus would help me keep track of my disease symptoms. |
|  | Health | Using DiaFocus helps me reduce my disease symptoms |
|  | Efficiency | Using DiaFocus helps me to reduce disease symptoms faster and more efficiently |
|  | Quality | Using DiaFocus improves the quality of my treatment. |
|  | Safety | Using DiaFocus reduces the risk of me having complications due to my diabetes. |
| **Effort Expectancy** | Usability | Overall, I would be satisfied with how easy it is to use DiaFocus |
|  | Understandable | My interaction with DiaFocus would be clear and understandable. |
|  | Learnability | It would be easy for me to learn to use DiaFocus |
|  | Ease of Use | I would find DiaFocus easy to use |
|  | Skillful | I would be skillful at using DiaFocus |
|  | Information Quality | The information I get from DiaFocus is clear and useful. |
|  | Interface Quality | The user interface is good when I use DiaFocus. |
|  | Pleasant | DiaFocus is comfortable to use. |
|  | Features | DiaFocus has the functionality I expect. |
| **Social Influence** | HCP | My [Health Care Professional] thinks that I should use DiaFocus. |
|  | Relatives | My family (e.g., my spouse) thinks that I should use DiaFocus. |
|  | Friends | My friends and colleagues think that I should use DiaFocus |
|  | Society | I am expected to use DiaFocus as a patient in the Danish healthcare system. |
| **Facilitating Conditions** | Resources | I would have the resources necessary to use DiaFocus, such as a smartphone. |
|  | Knowledge | I would have the knowledge necessary to use [system name]. |
|  | Support | Someone would be available to help with technical issues. |
| **Behavioral Intention** | Intent | I plan to use DiaFocus in the coming months. |
